# Learning Impact of a Virtual Brain Electrical Activity Simulator Among Neurophysiology Students: Mixed-Methods Intervention Study

**DOI:** 10.2196/18768

**Published:** 2020-12-30

**Authors:** Marko Henrik Björn, Jonne MM Laurila, Werner Ravyse, Jari Kukkonen, Sanna Leivo, Kati Mäkitalo, Tuula Keinonen

**Affiliations:** 1 School of Applied Educational Science and Teacher Education, Joensuu University of Eastern Finland Joensuu Finland; 2 Institute of Biomedicine, Integrative Physiology and Pharmacology University of Turku Turku Finland; 3 Turku University of Applied Sciences Turku Finland; 4 Department of Clinical Neurophysiology Turku University Hospital Turku Finland; 5 Faculty of Education University of Oulu Oulu Finland

**Keywords:** virtual simulation, electroencephalography, theoretical knowledge, neurophysiology, brain activity, psychomotor

## Abstract

**Background:**

Virtual simulation is the re-creation of reality depicted on a computer screen. It offers the possibility to exercise motor and psychomotor skills. In biomedical and medical education, there is an attempt to find new ways to support students’ learning in neurophysiology. Traditionally, recording electroencephalography (EEG) has been learned through practical hands-on exercises. To date, virtual simulations of EEG measurements have not been used.

**Objective:**

This study aimed to examine the development of students’ theoretical knowledge and practical skills in the EEG measurement when using a virtual EEG simulator in biomedical laboratory science in the context of a neurophysiology course.

**Methods:**

A computer-based EEG simulator was created. The simulator allowed virtual electrode placement and EEG graph interpretation. The usefulness of the simulator for learning EEG measurement was tested with 35 participants randomly divided into three equal groups. Group 1 (experimental group 1) used the simulator with fuzzy feedback, group 2 (experimental group 2) used the simulator with exact feedback, and group 3 (control group) did not use a simulator. The study comprised pre- and posttests on theoretical knowledge and practical hands-on evaluation of EEG electrode placement.

**Results:**

The Wilcoxon signed-rank test indicated that the two groups that utilized a computer-based electrode placement simulator showed significant improvement in both theoretical knowledge (Z=1.79, *P*=.074) and observed practical skills compared with the group that studied without a simulator.

**Conclusions:**

Learning electrode placement using a simulator enhances students’ ability to place electrodes and, in combination with practical hands-on training, increases their understanding of EEG measurement.

## Introduction

### Simulation

Simulators are devices that mimic the technical and physical aspects of real-life activities and are typically used in training environments where real-life practice is not viable [1]. Simulators are as closely correlated as possible to existing guidelines and protocols of the process they are designed to imitate [2]. Room-scale simulators have multiple restrictions that mostly derive from excessive costs and size, making them unattainable by most higher education settings. For teaching and learning purposes, where multiple students would require simultaneous access to simulator training, personal computer (PC)-based simulation is seen as a viable alternative [3]. The primary function of simulators in a teaching and learning setting is not just to facilitate practical skill acquisition, but also to foster theoretical understanding [4]. Learning via PC-based simulation is experiential learning where the teacher is not always present [5]. However, students who use simulators gain better self-confidence and are less anxious when confronted with the actual event of a simulator-trained process than students who have not used simulators [6,7].

Simulations have also been used in the teaching of electroencephalography (EEG) [8]. EEG is a simple method used to monitor brain electrical waves, most commonly as a diagnostic tool for epilepsy. In EEG, electrodes are placed on the scalp and biosignals generated by cerebral neurons, modified by electrical conductivity properties of the tissues, are recorded between the electrical source and the recording electrodes. This method is widely used in neurophysiological clinical diagnosis. In EEG, evoked potentials (visual, somatosensory, motor, auditory) are examples of the recorded brain responses. In addition, different tools for diagnosing sleep disorders, such as polysomnography, respiratory polygraphy, reflex studies (blink reflex and masseter reflex), electroretinography, and electroneuromyography are examined using EEG [9].

As in all educational sectors nowadays, simulation-based approaches are increasingly being utilized in biomedical and medical education; they are used to support student learning and clinical training [10], as well as training in medical procedures [1]. In health care education, simulations are used to improve clinical performance [11] and clinical reasoning [4]. However, learning practical skills (such as EEG electrode placement) requires hands-on exercises, and in biomedical laboratory science education these exercises are usually performed in a laboratory environment [12] rather than through practical training in hospitals. Unfortunately, due to the large number of students who need EEG training, hands-on practice sessions cause disruptions at hospitals. Limited equipment and laboratory staff resources have resulted in a situation where students have a severely constrained number of EEG placement practice sessions. For this reason, teachers tend to resort to lecturing about EEG placement, which usually results in a lack of readiness of students to do practical exercises with adequate guidance.

As a result, professional EEG laboratory staff are more involved in addressing the fundamental skills (eg, identifying the head measurement starting point) of EEG electrode placement rather than providing the holistic EEG experience that a clinical laboratory session should provide. Using PC-based simulations in the field of clinical neurophysiology could be helpful to train biomedical laboratory students in the method of EEG in both practical and theoretical perspectives [13,14]. Even though PC-based simulators have become very important tools in the field of education [15], the current simulator research revealed limited academic interest and no commercially developed PC-based EEG simulator exists to date. There are some commercially available EEG estimators, but there are no educational EEG simulators for the electrode placement system. For educational purposes, some EEG simulators exist mainly for identifying EEG activity. In general, it is believed that EEG simulators are an effective, user-friendly, and inexpensive method for learning EEG morphology and recognizing seizure activity [16]. Some companies delivering EEG measurement devices have developed EEG simulators for control purposes. Efforts are currently underway to develop an EEG results simulator for ensuring that simulation quality is relevant in EEG measurement [17].

### PC-Based Simulator for EEG Electrode Placement

Routine EEG examinations are implemented in clinical laboratories. Therefore, understanding the EEG recording system is very important for biomedical laboratory scientists working in clinical neurophysiology department at hospitals. In EEG, electrodes should be placed in the correct positions on a patient’s scalp so that the device measurement is reliable while being as comfortable as possible for patients.

In Finland, all biomedical technicians (biomedical laboratory scientists) and nurses have graduated from universities of applied sciences with studies in clinical neurophysiology. Although the details of the study curricula across universities may differ, they all contain a theoretical (ie, classroom) component followed by practical (ie, laboratory) training. There is consensus among lecturers from several Finnish universities and laboratory professionals that the EEG method skills (ie, the underlying theory for practical work with EEG equipment) of students entering the practical training is substandard. This places an additional burden on the laboratory professionals who administer the practical training and could lead to students not being able to successfully complete their neurophysiology studies. If this situation remains unresolved, universities may soon be producing graduates who require work-under-supervision conditions or further on-the-job training before being effective and efficient biomedical technicians.

To better prepare students for practical training, we have developed a PC-based EEG simulator for learning the EEG method and neurophysiology. Our EEG simulator is based on the routinely used 10-20 mapping system [18] for electrode positioning and presents a 3-dimensional model of a human head on which students practice the placement of EEG electrodes according to this system. Our simulator relies on feedback, both immediate and summative, as its primary catalyst for learning. Feedback refers to how close to the correct position trainees place EEG electrodes on the virtual head. Instant updates, as electrodes are placed, give players an opportunity to experience instantaneous response [19] regarding their electrode placement accuracy and an opportunity to learn by correcting their electrode positioning. In addition, our EEG simulator presents a summative scoring feedback immediately after the assignment. This satisfies student expectation because it is similar to traditional in-class learning situations where written feedback from the teacher for learning assignments and percentage grade feedback for exams are given postevent [20]. In our case, the summative feedback serves as an enabler for postsimulator debriefing [21], where students can process and strengthen their simulator learning events [22].

**Figure 1:**
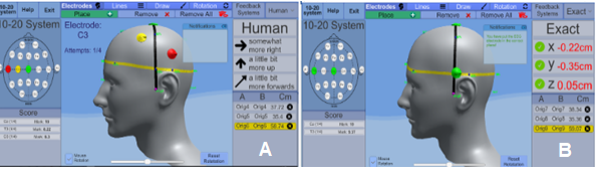
Electroencephalography simulator interface screenshots showing fuzzy (A) and exact (B) feedback systems.

The EEG simulator contains two feedback systems—exact and fuzzy—for users. The fuzzy logic system provides human-like feedback through linguistic variables (ie, words) as a way to define results without a precise answer, whereas the exact system gives an axis and magnitude metric of how far away from the correct location the placement is. For example, the fuzzy feedback system provides feedback such as “placement is a little too far left” or “placement is too low,” while the exact feedback system would give “placement is 6.8 mm to the left” or “placement is 32.8 mm too low.” Figure 1 shows two instances of the main interactive view of the EEG simulator application, illustrating (on the right side of each image) the fuzzy and exact feedback systems. A more detailed technical explanation of the application and its implementation was presented in our previous study [8], in which students’ perceptions on feedback mechanisms were examined; students initially favored the fuzzy feedback system, but after a period of practicing and improving electrode placement precision, they wanted to know the exact accuracy of their placements (ie, the exact feedback system).

The main purpose of this study was to extend our earlier research by exploring how the introduction of a PC-based EEG simulator in a higher education neurophysiology course would enhance students’ acquisition of practical skills. This study was also cognizant of the importance of theoretical neurophysiology knowledge and therefore also investigated the impact of EEG simulator use on theoretical knowledge. The following hypotheses were developed for this study:

- Hypothesis 1: students utilizing the EEG simulator would show greater improvement in theoretical knowledge that those who did not study using the simulator.
- Hypothesis 2: students practicing with the EEG simulator’s fuzzy feedback system would show better hands-on skills for electrode placement than those practicing with the exact feedback system and those not using the EEG simulator at all.

## Methods

### Context of the Study

**Figure 2:**
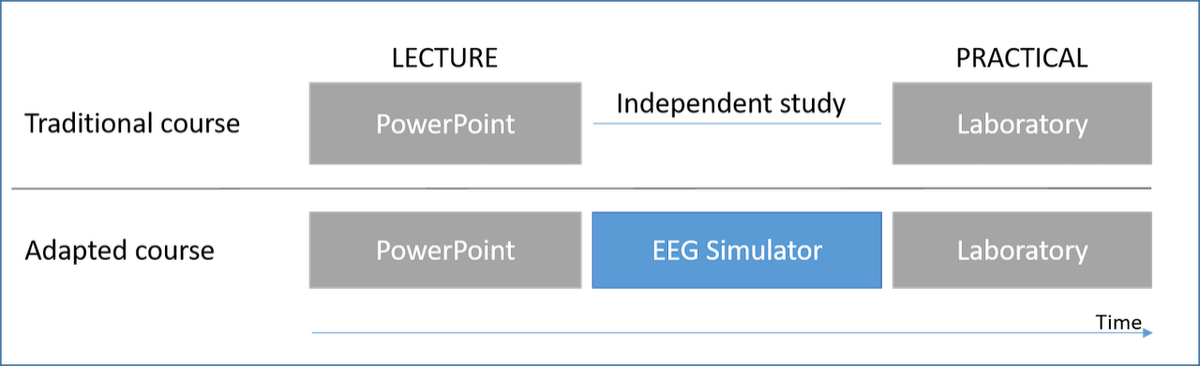
Traditional and adapted neurophysiology course. EEG: electroencephalography.

The context of this study was a clinical neurophysiology course that had an identical implementation of EEG training at two universities of applied sciences in southern Finland. The first author was the teacher of the training session at both universities. Figure 2 depicts a high-level comparison between the traditional course setup and our newly adapted multimodal study approach containing additional PC-based EEG simulations.

The clinical neurophysiology course included EEG and electromyography methods and evoked potentials; this study focused only on the EEG method. The course implementation and data collection were carried out in the autumn semester of 2018 at one university and in the spring semester of 2019 at the other university. Using the new multimodal study approach, both courses began with a prerecorded online lecture about the basics of the EEG method and neurophysiology. We provided supplementary study material on subject-specific issues for independent studies. All students had access to this material via an e-learning platform and were encouraged to review it at their discretion. After online lectures at the beginning of the course, students had three consecutive days to practice with the simulator for 2 hours/day under teacher guidance. Two weeks afterward, students attended the practical hands-on sessions carried out in the laboratory, where students were able to work with a real EEG hardware system to place the EEG electrodes on the scalp of a coworker’s head. The final laboratory exercises were carried out at the department of clinical neurophysiology in the respective university hospitals.

### Research Design and Data Collection

**Figure 3:**
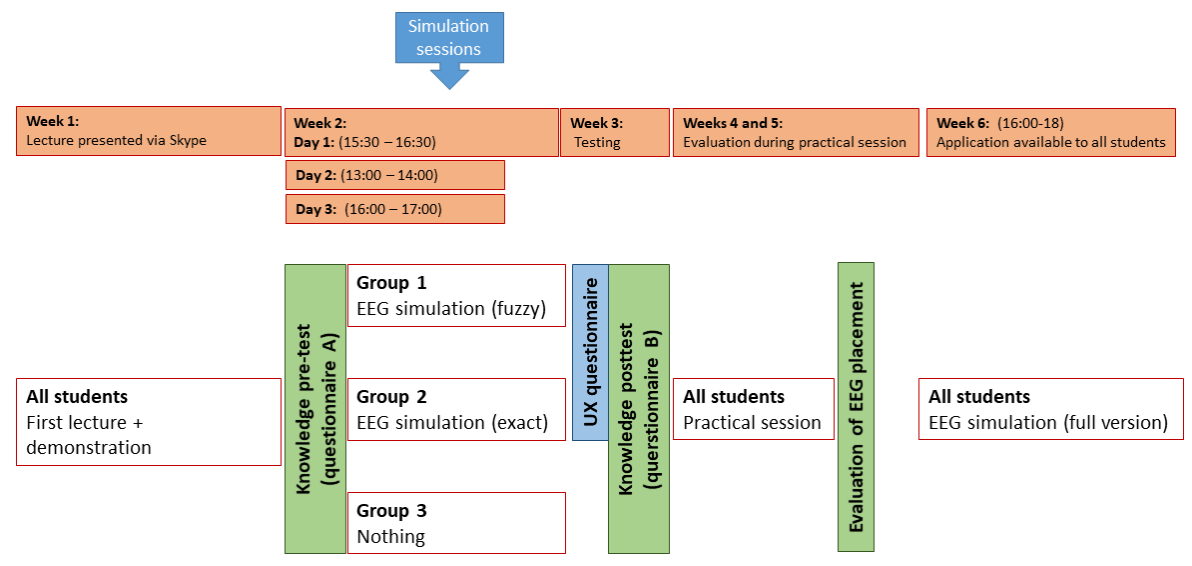
Time schedule for the study and data collections. EEG: electroencephalography; UX: user experience.

All students in the clinical neurophysiology course at both universities were invited to participate in the study during their respective introductory sessions, and a total of 35 students—10 male and 25 female students aged 20 to 23 years—volunteered to participate. Figure 3 shows the timeline for student activities and data collection during a 5-week data collection period. This study was piloted with 10 students before the actual study was conducted.

All students participated in the same prerecorded online introduction, and students’ baseline theoretical knowledge was determined using a pretest (questionnaire A). After this pretest, students were randomly assigned into 3 groups: (1) group 1 studied using an EEG simulator with fuzzy feedback, (2) group 2 studied using an EEG simulator with exact feedback, and (3) group 3 (control group) studied without the EEG simulator. All groups were comprised of 11 to 12 students. After 3 guided simulation sessions for groups 1 and 2 and an independent study period for group 3, a posttest (questionnaire B) was administered to determine possible knowledge improvement. The pretest and posttest (Multimedia Appendix 1) both contained 8 questions—4 neurophysiology theory questions and 4 questions regarding the EEG method for electrode placement. The tests contained different questions to avoid the possible impact of the pretest on the posttest. To ensure that the pre- and posttest questions were valid, we selected questions from neurophysiology training guidelines. Furthermore, to safeguard that the questions were also of a suitable difficulty level, we asked the clinical teacher at the university hospital to evaluate them beforehand. During the 2-week simulation periods with the EEG simulator, students in group 3, who did not have access to the EEG simulator, were asked to study the supplementary learning materials shared on the EdX e-learning platform. Students in groups 1 and 2 also had access to these materials during our study. Students in groups 1 and 2 (who used the simulation) were instructed to keep a diary of their experiences with the simulator and asked to fill in a user experience (UX) questionnaire after using the device.

To test our hypotheses, a pretest–posttest design was implemented to characterize the effect of EEG simulations on theoretical knowledge about EEG methods and neurophysiology. The practical skill of EEG electrode placement was examined with hands-on sessions carried out in a real laboratory environment where two teachers evaluated students’ EEG electrode placement skills according to EEG guidelines [10].

In order to give all students the opportunity to use the simulator, the full version of the simulator was shared with all study participants after the practical evaluations. This simulation version contained both feedback systems. In this way, each student in the study had an opportunity to learn from EEG simulation.

To help the simulator designers, UX questionnaire data were collected from everyone within 3 weeks of using the simulator. These data provided insights to help refine the simulation process.

The usefulness of the EEG simulator and its feedback systems in practice was evaluated by observing students’ accuracy on EEG placements during hands-on laboratory sessions. In addition, students were guided to keep a learning diary on their experiences and feelings during their studies.

### Evaluation of EEG Electrode Placement Skills

During the students’ practical training of EEG electrode placement and measurement, their work was observed and evaluated. An expert clinical neurophysiologist/clinical teacher and the neurophysiology course instructor (SL and MHB) used two sets of assessment guidelines, based on the consultations with several other practicing clinical neurophysiologists. The first set focused on the students’ ability to identify and measure skull dimensions, while the second set of guidelines evaluated student EEG electrode placement accuracy. The measurement assessment guidelines included the following: (1) identifying the nasion and inion points of the head, (2) measuring the distance between the nasion and inion electrode points, (3) identifying the right and left preauricular point positions on the head, and (4) measuring the head circumference.

**Figure 4:**
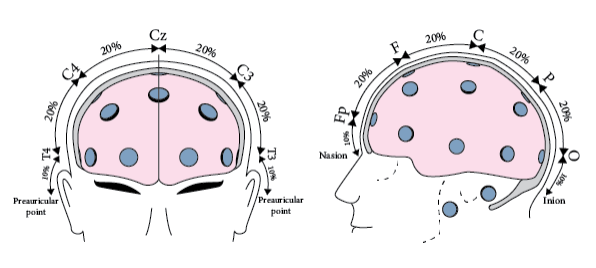
Frontal and sagittal plane views of the electroencephalography electrode placement points in the 10-20 electrode measurement system [18].

The identification and measuring skills are important because they give the precise location of the central electrode (C_z_), as well as the other electrodes’ nasion-inion line. Figure 4 shows the target points from the first set of assessment guidelines on how to set up electrodes on the skull.

The placement assessment guidelines included the following items for evaluation: (1) accuracy of EEG electrode placement on the skull; (2) technique for measuring distance between the 3 z-points on the skull (C_z_, F_z_, P_z_); (3) how to measure C_z_ electrode position on the center of the skull; (4) how to measure O_1_, O_2_, F_p1_, and F_p2_ electrode positions on the skull; (5) what system was used in the EEG measurement; (6) how to identify right and left preauricular points on the head; and (7) result (cm) from the head circumference measurement.

**Figure 5:**
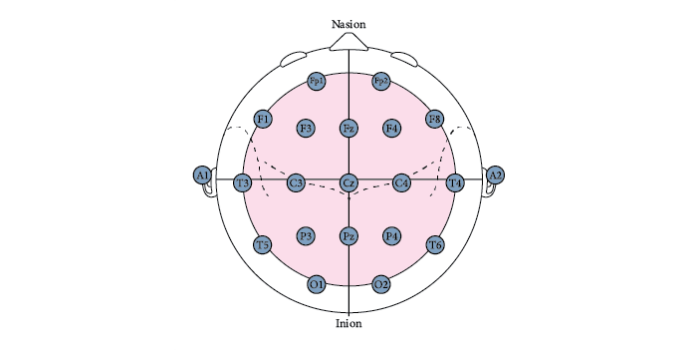
Electrode positions on the skull using the 10-20 electrode electroencephalography measurement system [18].

The accurate placement of all electrodes is critical to obtaining reliable EEG data. If the electrodes are misplaced, data from an EEG scan become unrealistic and even erratic, making any diagnosis impossible. Figure 5 shows the exact placement of electrodes according to the 10-20 system.

### Data Analysis

Pre- and posttest answers were analyzed statistically using the SPSS software package (IBM Corp). To compare the quantitative results of pre- and posttests between groups, a Wilcoxon signed-rank test was performed. Learning diaries were analyzed by content analysis using ATLAS.Ti (ATLAS.ti Scientific Software Development GmbH) and a deductive reasoning approach. These deductions allowed the clarification of students’ written answers. Practical part observations were carried out using guidelines that were created for all neurophysiology laboratories using EEG measuring methods [23].

## Results

This study was comprised of 35 students. Table 1 shows the results from the pre- and posttests. Students who used the exact feedback mode in the EEG simulation scored slightly higher in their knowledge of the EEG method after the simulation than those who used the fuzzy feedback mode. Students who studied using the EEG simulator exhibited greater knowledge of the EEG method than students who did not use simulation. Only students in group 3 (students who did not use the EEG simulation) showed a significant improvement in their knowledge of neurophysiology (Z=1.79, *P*=.074) within the group. Group 3 also had the lowest baseline test scores of knowledge of neurophysiology. Groups 1 and 2 did not show significant improvement in their knowledge of neurophysiology after using the EEG simulator.

**Table 1:** Students’ pre- and posttest knowledge scores within the study.

		Students’ knowledge test scores					
		Group 1 (fuzzy feedback)		Group 2 (exact feedback)		Group 3 (no simulation)	
Subject area and test		Mean (SD)	Median	Mean (SD)	Median	Mean (SD)	Median
**Neurophysiology**							
	Pretest (questionnaire A)	11.30 (1.70)	11.50	11.58 (1.62)	11.50	10.62 (1.85)	10.00
	Posttest (questionnaire B)	12.20 (1.99)	13.00	12.80 (2.04)	13.00	13.17 (1.83)	13.00
**EEG^a^ method**							
	Pretest (questionnaire A)	9.20 (1.55)	10.00	8.17 (2.69)	8.00	9.62 (1.98)	10.00
	Posttest (questionnaire B)	11.80 (2.66)	12.50	12.40 (2.27)	12.50	10.17 (2.32)	10.50

^a^EEG: electroencephalography.

At the beginning of the study, after an online prelecture and based on the results of the pretest (questionnaire A), there was no significant difference in the students’ basic knowledge of the EEG method between the groups (Table 1). When comparing the mean test scores for the EEG method, a significant improvement in knowledge among students of all groups between pre- and posttests was found (Z=3.02, *P*=.003). When knowledge of the EEG method within the groups was compared, students who used the exact feedback system in the EEG simulation showed the greatest increase during the study (Z=2.32, *P*=.021). In addition, students who used the fuzzy feedback system in the EEG simulation showed significantly higher knowledge of the EEG method in the posttest compared with the pretest (Z=2.20, *P*=.028). Only students who did not use the EEG simulation in their studies failed to show a significant improvement in their scores of knowledge of the EEG method from the pretest to the posttest (*P*=.892). A similar trend was seen in the median test scores, where the scores improved 2.50, 4.50, and 0.50 between the pre- and posttest for the fuzzy feedback group, exact feedback group, and no simulation group, respectively. Students who studied using the EEG simulation knew better how and where the EEG electrodes should be placed on the skull. However, it was also clear that these students still needed teacher support in learning the correct placements of the EEG electrodes even after the simulation.

In addition, students who used the EEG simulator were asked to write about the simulation experience—including benefits, disadvantages, motivations, and novelty—and their diary entries suggested that the EEG simulation influenced their learning, as shown in the following excerpts:

The simulation was helpful in learning the right electrode positions.

S11

The EEG simulation made it easier to visualize the positions of the electrodes. It explained a lot about how to do it in practice. The simulation did not help outline other things about neurophysiology.

S1

Learning motivation is enhanced by the fact that the instructions, for example with the help of pictures, are clear. The trial-and-error approach to learning that the simulator brings, however, does not necessarily increase motivation.

S12

Based on the learning diaries, 45.7% (16/35) of the students indicated that PC-based simulations are generally useful in education. In addition, one-half (18/35, 51.4%) of the students indicated that they would be ready to use their own time to practice the EEG method and electrode placements using a PC-based simulator. However, several students mentioned that hands-on skill practice cannot be totally replaced by virtual simulation, as those skills need to be practiced in the laboratory environment as well.

I was positively surprised by the introduction of an EEG placement simulator, and the initial idea that this could provide some help with learning. Of course, simulations are no substitute for practice.

S1

The electrode placement during simulator utilization sessions was continuously improving and I was getting faster at placing electrodes every time I restarted. The simulation provides a good foundation before the actual hands-on practical electrode placement session. However, there is very little theory in the simulation.

S11

Unfortunately, some absenteeism in the evaluation of practical skills brought small changes in the number of participants. Eight students from group 1 (fuzzy feedback), 12 students from group 2 (exact feedback), and 12 students from group 3 (no simulation) completed the practical EEG electrode placement session. Since placement accuracy is vital to EEG diagnosis, students were evaluated as being “successful” or “unsuccessful” in their task. Only students who placed all electrodes accurately were considered to have successfully completed the task. Table 2 presents the number of students from each group who successfully or unsuccessfully completed the electrode placement task.

**Table 2:** Results from practical evaluations of electrode placement.

	Group 1 (fuzzy feedback; n=8)		Group 2 (exact feedback; n=12)		Group 3 (no simulation; n=12)	
Measurement	Successful	Unsuccessful	Successful	Unsuccessful	Successful	Unsuccessful
C_z_ electrode position	8	0	12	0	9	3
O_1_, O_2_, F_p1_, and F_p2_ electrode positions	8	0	10	2	9	3
10-20 EEG^a^ system	8	0	12	0	6	6
Right and left preauricular points	8	0	12	0	9	3
Head circumference	8	0	11	1	7	5

^a^EEG: electroencephalography.

Evaluation through observation of the practical placements confirmed the results of the pre- and posttests. Students who studied using the EEG simulator showed greater skills at placing the electrodes. When comparing the results of groups 1 (fuzzy feedback) and 2 (exact feedback), students who used simulation with the fuzzy (human-friendly) feedback system performed the best in practical placements. These students demonstrated a superior placement accuracy compared with the students from group 2, the exact feedback system, who had problems in O_1_, O_2_, F_p1_, and F_p2_ electrode position measurements, as well as in head circumference measurement. Students who did not use the simulator (group 3) had difficulty overall in the EEG electrode placement. They managed to complete the practical part of the task (ie, the EEG electrode placement) only with the support of the teacher.

Some students in group 3 (no simulation group) expressed that placing the EEG electrodes on their coworker’s head was challenging because electrodes were moving on the scalp. Some students in group 2 (exact feedback group) had problems getting O_1_, O_2_, F_p1_, and F_p2_ electrodes to work properly. However, this was a consequence of an inexact placement of EEG electrodes. Students using the fuzzy feedback system (group 1) indicated that the EEG simulation helped them to better remember the placement of electrodes and distances from each other on the head. On the other hand, these students commented that studying by simulation was not the same as placing the electrodes in a practical setting because the simulation did not include hands-on work, as seen in the following excerpt:

The simulation helped, but it was good to see and experience how the electrode placement was actually done… which tools are used and how to measure them. Electrodes placement vary from laboratory to laboratory.

S16

## Discussion

### Principal Findings

The results confirm the hypothesis that studying using a simulator provides additional support for learning the EEG method and showed a positive influence in students’ learning of neurophysiology. The increase in knowledge may at least partly be a consequence of increased motivation of those students that used the simulation during their studies. The results indicate that simulation with a logical (human-friendly fuzzy feedback) system has a more positive impact on practical skills, but the exact feedback simulation is an important tool from the theoretical knowledge development point of view. On the other hand, the theoretical knowledge of those students who did not use simulation increased the most, especially concerning the basics of neurophysiology, thereby refuting our first null hypothesis. This can be explained by the fact that those students who did not use the simulator may have had more time to concentrate and to study the theoretical supplementary materials provided in the course. We noted that the diary entries of the students using the simulator did not mention any additional engagement with theory learning materials to augment their practice of the EEG method. It may also be that because the nonsimulator students knew that they were part of a study that was measuring learning, they made extra efforts to learn the material. However, these students were not able to achieve all additional learning that the exact and fuzzy simulator groups enjoyed. Learning hands-on skills requires some instruments, and for the EEG method, this learning was enhanced with PC-based simulation.

This study revealed that for students using the EEG simulator, their knowledge of the EEG method increased more than their knowledge of neurophysiology. The insignificant improvement in their knowledge of neurophysiology through use of EEG simulation was also due to the fact that the simulator did not require the students to be immersed in theoretical physiology-related material. This is in line with the results of Jaakkola et al [12], which suggest that the best learning results are gained using a multimodal learning approach, where virtual simulations are done together with practical exercises. Nevertheless, the modest improvement in neurophysiology knowledge among students using the simulator resulted from the requirement to learn the 10-20 system, which links to some aspects of neurophysiology theory.

Although students preferred the fuzzy feedback mode, the purposeful nature of their encounters with the learning material to test our second hypothesis explains why both the fuzzy and exact feedback systems led to significant improvement in knowledge of the EEG method. Students who did not use the simulator did not show significant improvement in their knowledge of the EEG method simply because they had no means of developing a practical frame of reference for electrode placement. This result is in line with that of Miller et al [16], who used a different EEG simulator, albeit without an electrode placement feature.

A further study by Bottomley et al [24] indicated that visualization increases the enjoyment of learning, making students more susceptible to learning. Although we did not study learning strategies, this observation of visual learning cannot be considered a coincidence, but it requires further study.

The learning diaries were only written during the simulation period. In other words, the students had no exposure to practical sessions at the time of keeping their diaries and the diaries were returned before the practical sessions. Students realized early on that a PC-based simulator could supplement hands-on training but could not replace it. It is clear that PC-based simulations cannot replace teachers themselves, as teachers are needed to familiarize students with new virtual learning environments. Improved technology only provides new tools for education but does not have an effect on the studied learning content [25]. Students’ comments in diaries indicated that less than half of the students believed that the EEG simulator might be useful, but the results clearly indicated that the simulator significantly influenced their learning. Furthermore, half of the students would be willing to invest their own time into using the simulator, meaning that the other half would require further encouragement, for instance through continued teacher oversight and guidance.

The students who used the simulator were more pragmatically engaged with the learning material throughout and therefore more inclined to remember all the steps when conducting the practical hands-on task. These research results support the notion that the use of PC-based simulations in education should be supported and encouraged.

### Conclusions and Future Work

In this study, we used an experimental research design to understand what influence a PC-based EEG simulator could have on both theoretical knowledge and practical skills among higher education biomedical laboratory science students. By measuring the learning outcomes, we were able to gauge whether the introduction of a PC-based simulator could better prepare students for practical hands-on sessions.

Although our study raised some reliability concerns due to a limited number of participants across 3 experimental groups, we remain confident that utilizing a PC-based EEG csimulator in neurophysiology classes gives students more opportunities and increases motivation to learn practical EEG placement. It did not, however, appear to improve learning of neurophysiology theory. It was also clear that students recognized the value of hands-on work when it comes to learning practical skills. Students strongly believed that the actual handling of EEG equipment is invaluable in learning how to use it, leading us to recommend that simulator training should supplement hands-on training and never attempt to replace it. Using a teaching strategy that complements theory lessons with PC-based simulator practice proved to significantly enhance practical learning of the EEG method among higher education biomedical laboratory science students, making students better prepared for hands-on training. We hereby postulate that using PC-based EEG simulators in courses that include other electrical medical devices, such as respirators and electrocardiographs, will also improve practical application knowledge of these devices.

This study completed a second design-develop-test-evaluate iterative cycle of an ongoing endeavor to improve neurophysiology competence among university students in the applied sciences. Future work will include minor revisions to some of the EEG simulator features and a longitudinal study to examine the long-term learning effects of using our simulator. More immediate efforts have gone into developing a mobile augmented reality application to complement the neurophysiology theory learning material. We are exploring whether such an application would stimulate enough intrinsic motivation to engage with the theoretical part of the course, thereby also improving conceptual understanding.

## Appendix

Multimedia Appendix 1 Pretest (questionnaire A) and posttest (questionnaire B).

## Abbreviations

EEG: electroencephalography
PC: personal computer
UX: user experience
