# Supplementary material for: Learning Impact of a Virtual Brain Electrical Activity Simulator Among Neurophysiology Students: Mixed-Methods Intervention Study
Source: JMIR Serious Games. 2020 Dec 30;8(4):e18768. doi: 10.2196/18768 (PMC7806441; doi:10.2196/18768)
Supplement: Multimedia Appendix 1 [file games_v8i4e18768_app1.docx]

Multimedia Appendix 1

The pre- (A) and post- (B) tests evaluated the following subjects and contained 8 specific multiple-choice questions:

Pre-test (A):

Neurophysiology:

| What are the basics of clinical neurophysiology? |
| --- |
| What does the EEG measure? |
| What kind of activations can be used in the EEG? |
| What is latency? |
| What are the main EEG artifacts? |
| What is meant by averaging in a neurophysiology test? |
| What may affect the signal amplitude in the EEG method? |
| What is important in EEG electrode placement in practical study? |

Post-test (B):

EEG

| Do most of the EEG (80%) recording signals lead to a diagnosis? |
| --- |
| What is the ten-twenty EEG electrode placement system? |
| When people are attentive to an external stimulus or are thinking hard about something, the alpha rhythm is replaced by what? |
| What is the EEG pattern in REM sleep? |
| What is the most prominent EEG wave in an awake, relaxed adult man whose eyes are closed? |
| Please describe the ten-twenty electrode system based on the International Federation recoding system after nasion and inion measurements. |
| Please explain the naming in the ten-twenty system. |
| What is the basic unit of resistance? Choose the right alternative. |
